# Diagnosis of Breakthrough Fungal Infections in the Clinical Mycology Laboratory: An ECMM Consensus Statement

**DOI:** 10.3390/jof6040216

**Published:** 2020-10-11

**Authors:** Jeffrey D. Jenks, Jean-Pierre Gangneux, Ilan S. Schwartz, Ana Alastruey-Izquierdo, Katrien Lagrou, George R. Thompson, Cornelia Lass-Flörl, Martin Hoenigl, European Confederation of Medical Mycology (ECMM) Council Investigators

**Affiliations:** 1 Division of General Internal Medicine, Department of Medicine, University of California San Diego, San Diego, CA 92103, USA; jjenks@ucsd.edu; 2 Division of Infectious Diseases and Global Public Health, Department of Medicine, University of California San Diego, San Diego, CA 92103, USA; 3 Clinical and Translational Fungal-Working Group, University of California San Diego, La Jolla, CA 92093, USA; 4 Univ Rennes, CHU Rennes, Inserm, EHESP, Irset (Institut de Recherche en Santé, Environnement et Travail)—UMR_S 1085, F-35000 Rennes, France; jean-pierre.gangneux@univ-rennes1.fr; 5 Division of Infectious Diseases, Department of Medicine, Faculty of Medicine and Dentistry, University of Alberta, Edmonton, AB T6G 2R3, Canada; ilan@ualberta.ca; 6 Mycology Reference Laboratory, National Centre for Microbiology, Instituto de Salud Carlos III. Majadahonda, 28222 Madrid, Spain; anaalastruey@isciii.es; 7 Department of Microbiology, Immunology and Transplantation, KU Leuven, 3000 Leuven, Belgium; katrien.lagrou@uzleuven.be; 8 National Reference Centre for Mycosis, Department of Laboratory Medicine, University Hospitals Leuven, 3000 Leuven, Belgium; 9 Division of Infectious Diseases, Department of Internal Medicine and Department of Medical Microbiology and Immunology, UC-Davis Medical Center, Sacramento, CA 95817, USA; grthompson@ucdavis.edu; 10 Division of Hygiene and Medical Microbiology, Medical University of Innsbruck, 6020 Innsbruck, Austria; cornelia.lass-floerl@i-med.ac.at; 11 Section of Infectious Diseases and Tropical Medicine, Department of Internal Medicine, Medical University of Graz, 8036 Graz, Austria

**Keywords:** breakthrough invasive fungal infections, invasive candidiasis, invasive mold infections, endemic mycoses, diagnostics

## Abstract

Breakthrough invasive fungal infections (bIFI) cause significant morbidity and mortality. Their diagnosis can be challenging due to reduced sensitivity to conventional culture techniques, serologic tests, and PCR-based assays in patients undergoing antifungal therapy, and their diagnosis can be delayed contributing to poor patient outcomes. In this review, we provide consensus recommendations on behalf of the European Confederation for Medical Mycology (ECMM) for the diagnosis of bIFI caused by invasive yeasts, molds, and endemic mycoses, to guide diagnostic efforts in patients receiving antifungals and support the design of future clinical trials in the field of clinical mycology. The cornerstone of lab-based diagnosis of breakthrough infections for yeast and endemic mycoses remain conventional culture, to accurately identify the causative pathogen and allow for antifungal susceptibility testing. The impact of non-culture-based methods are not well-studied for the definite diagnosis of breakthrough invasive yeast infections. Non-culture-based methods have an important role for the diagnosis of breakthrough invasive mold infections, in particular invasive aspergillosis, and a combination of testing involving conventional culture, antigen-based assays, and PCR-based assays should be considered. Multiple diagnostic modalities, including histopathology, culture, antibody, and/or antigen tests and occasionally PCR-based assays may be required to diagnose breakthrough endemic mycoses. A need exists for diagnostic tests that are effective, simple, cheap, and rapid to enable the diagnosis of bIFI in patients taking antifungals.

## 1. Introduction

Invasive fungal infections (IFIs) cause significant morbidity and mortality, particularly in patients with compromised immune systems, such as patients with underlying hematologic malignancies, hematopoietic stem cell transplant recipients (HSCT), solid organ transplant (SOT) recipients, and others who are critically ill in intensive care units (ICUs). In many of these patients, antifungal prophylaxis and/or early empirical treatment are used during the greatest period of risk for IFI to decrease morbidity and mortality from these infections. Still, some patients will develop a breakthrough IFI (bIFI) [1,2], defined as any IFI that occurs during adequate exposure to an antifungal agent, including from fungi outside the spectrum of activity of the antifungal agent, as recently defined in detail by the European Confederation for Medical Mycology (ECMM) and Mycoses Study Group Education and Research Consortium (MSGERC) consensus criteria [3]. Of note, initial improvement of clinical, radiological or mycological signs of IFI is an added requirement to differentiate breakthrough IFI from refractory IFI in those receiving targeted or pre-emptive therapy [3].

The diagnosis of bIFI can be challenging. Overall, while they remain the cornerstone of IFI diagnostics, culture-based approaches are limited by low sensitivity in patients exposed to antifungals, and delays in diagnosis are common [4]. In addition, conventional biomarkers that have become the mainstay of diagnosis of IFIs and specifically on invasive aspergillosis, such as 1,3-β-d-glucan (BDG) and galactomannan (GM), respectively, are negatively influenced in patients receiving mold-active prophylaxis or treatment [5,6,7,8,9].

Here, we review the literature on the diagnosis of bIFI, including conventional diagnostics such as culture, serologic tests, nucleic-acid based assays, and other modalities for the diagnosis of bIFI from both yeasts and molds. Lastly, we provide consensus recommendations on behalf of the ECMM.

## 2. Materials and Methods

Executives of the ECMM selected a group of authors based on content expertise, including individuals involved with the ECMM either as council members, fellows, or via the worldwide guideline initiative, with expertise in the diagnosis of yeast, mold infections, and endemic mycoses. ECMM is the umbrella organization of 28 national mycological societies, comprised of one delegate from each of the 28 nations forming the ECMM council (www.ecmm.info) [10,11].

According to their expertise authors were divided into three groups and assigned to breakthrough IFI caused by yeasts (*n* = 3), molds (*n* = 3), and endemic mycoses (*n* = 2). The authors searched PubMed for relevant English language articles on clinical studies of antifungal prophylaxis and treatment through July 2020. Search terms included “diagnosis”, “antifungal prophylaxis”, “antifungal treatment”, and “breakthrough fungal infection”. Study selection and data extraction were performed separately for yeast infections, mold infections, and endemic mycoses, there was no strict methodical process for the literature search, and the inclusion or exclusion of studies was at the discretion of the authors of these sections. There was no intent to grade the quality of the studies. Authors then drafted a consensus statement for diagnosis of breakthrough IFI.

A draft proposal for definitions was developed and sent out by the president of the ECMM to all ECMM council-members for critical revision, comments and suggestions, which were implemented into the final draft.

## 3. Consensus Recommendations

### 3.1. Diagnosis of Breakthrough Infections Caused by Yeasts

#### 3.1.1. Conventional Diagnostics

Clinical samples analyzed when an invasive yeast infection is suspected depends on the suspected location(s) of fungal infections and typically include blood, urine, cerebrospinal fluid, or tissue biopsies for deep or systemic infections. Skin scrapings, shaved nail or hair, vaginal secretions, and swabs allow the detection of superficial infections [12].

Fungal culture is one of the primary lab-tests used to diagnose bIFI as it allows the identification of the fungal pathogen and supports antifungal susceptibility testing. Species identification and antifungal susceptibility profiles can help guide antifungal treatment. The most commonly used culture media are Sabouraud dextrose and malt extract agar plates. Additional specialized media such as chromogenic agar allow the separation of similar-looking colonies in cultures with mixed growth of more than one yeast genus or species and the direct identification of some *Candida* species [13,14]. The missed diagnosis of a mixed yeast infection is of particular significance for breakthrough fungal infections especially if the missed yeast genus/species is resistant to the antifungal drug in use. Utility of chromogenic agar as primary isolation medium may be of particular help in this respect. Matrix-assisted laser desorption ionization–time of flight mass spectrometry has become a standard tool for the accurate, rapid, and economical identification of pathogens in the clinical diagnostics laboratory [15].

Microscopic examination of a primarily sterile site can determine whether or not the infection is due to a fungus and differentiate between fungal colonization and IFI [14,16]. Microscopy, however, cannot determine the specific cause of infection. While Gram stain lacks optimal sensitivity, fluorescent brighteners (Calcofluor white, or Blankophor), which bind to chitin in the fungal cell wall, are a rapid means of scanning samples for fungal structures, and enhance morphology assessment [17].

Interpretation depends on the type of sample investigated [14,16]. Yeasts obtained from non-sterile body sites, like the oropharynx or airways, may be part of the mycobiota or may be the causative agent of the infection. Hence, global assessment of the patient, which includes consideration of the history and physical examination as well as the microbiological findings, is of utmost importance to determine if the recovered yeast represents colonization or is causing IFI. Appearances may be highly characteristic of certain infections, such as India ink in cerebrospinal fluid, which can identify encapsulated yeast genera such as *Cryptococcus* spp. The microscopic detection of typical budding yeast cells, pseudohyphae, and/or true hyphae in samples obtained from otherwise sterile sites is indicative of fungal infections.

#### 3.1.2. Serology Including Antigen-Based Tests

Non-culture-based methods are increasingly used in clinical practice for the management of patients at high risk of fungal infection and can help reduce the time to diagnosis and allow for timely initiation of antifungal treatment. Antibody-based techniques are based on detecting circulating antigens in different body fluids. Enzyme-linked immunosorbent assay (ELISA) kits for detection of *Candida* mannan antigen are commercially available to detect Candida in serum samples for the diagnosis of invasive candidiasis (Platelia *Candida* Antigen, Bio-Rad Laboratories, Marnes-la-Coquette, France). When used in combination with anti-*Candida* mannan antibodies (Platelia *Candida* Antibody, Bio-Rad), this combination of serology tests has demonstrated good sensitivity (83%) and specificity (86%) [18]. However, the sensitivity of both mannan and anti-mannan vary for different *Candida* species, with lower sensitivity for *C. parapsilosis* and *C. krusei* [18]. Of note, the number of studies evaluating these assays is limited, and whether performance of mannan/anti-mannan is impacted by antifungal agents remains unknown.

The presence of 1,3-β-d-glucan (BDG) in serum can be used to diagnose some fungal infections (including *Candida* but not *Cryptococcus*). Since it is present in the cell wall of several fungal species [19], a positive result is not specific for invasive candidiasis. While the vast majority of studies to date evaluated the Fungitell^®^ assay (Associates of Cape Cod Diagnostics, MA, USA), other commercial test are available which may show similar performance; however, optimal universal cut off values for non-Fungitell tests are still lacking [20,21]. Sensitivity and specificity for diagnosing invasive candidiasis are both around 80% [22,23], but false positive results have been described [24], in particular in conditions associated with fungal translocation in the gut such as sepsis or advanced liver cirrhosis [25,26]. BDG results should, therefore, be carefully evaluated and always interpreted with other clinical data. Serum BDG may be a useful tool for diagnosing bIFI; however, similar to other diagnostic tests, reduced sensitivities have been observed in the presence of antifungal prophylaxis or treatment [27,28].

*Cryptococcus* antigen can be detected by a lateral flow assay (LFA; CrAg Immuno-Mycologics [IMMY], Norman, OK, USA), or via latex-agglutination (CryptoPlus assay, Bio-Rad). The LFA has high sensitivity (98–100%) and specificity (97–100%) in serum, plasma, cerebrospinal fluid, and urine and is the recommended biomarker for the diagnosis of cryptococcosis [29]. This assay has been extensively evaluated and it has been included in the “essential in vitro diagnostic list” of the WHO [30] and, thus, is recommended by the WHO for the screening and diagnosis of patients at risk for cryptococcal infection [31]. An ELISA kit is also available but is less commonly used due to a comparative performance with the LFA and the advantage of the LFA being a true point-of-care test (POCT).

#### 3.1.3. Nucleic Acid-Based Assays/Others

Using molecular tools, it is possible to diagnose and identify yeasts directly from clinical samples (including blood, serum, plasma, other sterile fluid, bronchoalveolar lavage, and tissues) and to rapidly identify the species attributed for positive blood cultures during bloodstream infections.

A large number of commercial and in-house targeted (simplex or multiplex) PCR assays with specific primers for various genetic sequences (18S rDNA, 28S rDNA, 5.8S rDNA, internal transcribed spacer regions and mitochondrial DNA) have been developed [32,33], including for infections from *C. auris* and its relatives, *C. haemulonii, C. duobushaemulonii*, and *C. pseudohaemulonii* [34].

Depending on the assay, multiplex panels or pan-fungal panels are available. In a meta-analysis of 54 studies with almost 5000 patients tested by blood-based PCR, pooled sensitivity and specificity for proven or probable invasive candidiasis vs. at-risk controls were 95% and 92%, respectively [35]. PCR plus blood culture, *Candida* PCR and, to a lesser extent, BDG testing, significantly enhanced the performance of PCR alone for the diagnosis of invasive candidiasis [28]. A recent and fully-automated assay combining internal transcribed spacer (ITS)2 region amplification and T2 magnetic resonance, the T2Candida^®^ Panel (T2Biosystems, Wilmington, MA, USA), has been developed. This assay detects three groups of *Candida* (*C. albicans/C. tropicalis*, *C. glabrata/C. krusei*/*C. bracarensis*, and *C. parapsilosis*/*C. orthopsilosis/C. metapsilosis*) in EDTA blood samples within 5 h and proved efficient for the diagnosis of candidemia and of intra-abdominal candidiasis [36,37,38].

While blood cultures lack sensitivity, they still represent the diagnostic gold standard for candidemia and molecular blood culture identification (BCID) panels provide precise and rapid identification of cultured pathogens. Results are obtained with minimal hands-on time compared to conventional methods like chromogenic media and biochemical identification, proteomic identification using MALDI-TOF MS or fluorescence in situ hybridization (FISH) assays. Two BCID kits offer different fungal panels: The GenMark Dx ePlex (Carlsbad, CA, USA) fungal pathogen panel (BCID-FP) rapidly detects 15 fungal targets (*C. albicans*, *C. auris*, *C. dubliniensis, C. famata, C. glabrata, C. guilliermondii*, *C. kefyr*, *C. krusei*, *C. lusitaniae*, *C. parapsilosis, C. tropicalis*, *Cryptococcus neoformans/gattii*, *Fusarium* spp., and *Rhodotorula* spp.) [39] and the Biofire FilmArray BCID-FP (BioMérieux, Marcy-l’Etoile, France) (*C. albicans, C. auris*, *C. glabrata*, *C. krusei*, *C. parapsilosis*, *C. tropicalis* and *Cryptococcus neoformans/gattii*) [40].

Metagenomic next-generation sequencing (mNGS), which analyzes the nucleic acids from a broad spectrum of mixed populations of microorganisms simultaneously, is a strategy that can potentially identify the causative pathogen when other strategies have failed, and has been used to diagnose cryptococcal meningitis in multiple studies [41,42] and case reports [43]. Still, more rigorous studies of this strategy in breakthrough yeast infections before the use of this technology becomes widespread.

#### 3.1.4. Consensus Recommendation

The cornerstone for diagnosing a breakthrough yeast infection relies on obtaining the isolate, most commonly with conventional culture methods. Prior antifungal treatment may confer a selection pressure for drug-resistant isolates. If a bIFI is diagnosed, the objective is to adapt the antifungal therapy to the species identified and to the in vitro susceptibility profile. The main steps of the diagnosis include the following:

- Direct examination of sterile samples is recommended for the proof of infection given the potential effect of antifungal therapy on fungal culture sensitivity. However, given limited sensitivity, a negative direct examination does not exclude infection.
- Once an isolate is grown, identification should be performed. Particularly in the case of a positive blood culture, molecular blood culture identification (BCID) panels provide precise and rapid identification.
- Antifungal susceptibility testing should be performed on invasive isolates to evaluate the activity of the current and alternative drugs.
- Non-culture methods of detection (serology and/or PCR) can be considered but the impact of antifungal therapy on their sensitivity has not been well-enough studied. Specificity is also a concern, especially with non-sterile samples, because highly sensitive molecular techniques can also reflect the presence of commensal yeasts.

### 3.2. Diagnosis of Breakthrough Infections Caused by Molds

#### 3.2.1. Conventional Diagnostics

To establish a diagnosis of “proven” invasive mold infection per consensus definitions by the European Organization for Research and Treatment of Cancer (EORTC) and the MSGERC [44] or the AspICU algorithm by Blot and colleagues [45], or other definitions for the ICU setting [46], histopathologic, cytopathologic, or direct microscopic examination of a specimen obtained by needle aspiration or biopsy must show hyphae with evidence of associated tissue damage, recovery of mold by culture from a normally sterile site, or a positive blood culture with compatible signs and symptoms of infection. Although microscopy (optimally using optical brighteners) and culture are the traditional cornerstones for the diagnosis of invasive mold infection, most diagnoses are not made from a sterile site and the diagnosis is determined to be “probable” or “possible” per EORTC/MSGERC criteria, or putative according to AspICU criteria. In the case of clinical suspicion of a bIFI more aggressive invasive diagnostic procedures, including biopsies, may be warranted, if possible.

Culture-based approaches have the potential to detect the causative fungal pathogen and antifungal resistance and are the gold standard for investigating bIFI by Mucorales, *Fusarium* spp., *Lomentospora* spp., and other rare molds for which reliable antigens and other diagnosis are not available [47,48,49,50]. However, it is not always possible to attempt to obtain tissue biopsy for culture from a sterile site due to the risk of excessive bleeding or clinical contraindications, especially in patients with thrombocytopenia. In addition, culture-based approaches lack sensitivity. Positive blood culture is seen in fusariosis, lomentosporosis, and IA caused by *Aspergillus terreus* [49,51], but very rare in other cases of invasive aspergillosis (IA) [52]. Most cases of IA in immunocompromised patients are not proven by EORTC/MSGERC criteria (i.e., culture from a sterile site or biopsy evidence of invasion): for example, in one large survey of HSCT recipients, only 11.5% of patients with IA met the criteria for proven infection [53]. The sensitivity of culture is imperfect, ranging from 30–60% from bronchoalveolar lavage fluid (BALF) [54], and is lower for diagnosing bIFI in patients taking antifungals. In a study of 53 patients with diagnosed IFI, of which 34/53 (64%) were on mold-active antifungal prophylaxis, 16/53 (30%) were diagnosed with “proven” infection, with a sensitivity of culture from BALF of only 3/16 (18.8%) [8]. Similar low sensitivities of culture have been described in other studies for patients on mold-active antifungals [8,55,56,57]. Thus, the diagnosis of bIFI in many cases may be missed if diagnosis is relied on conventional diagnostics such as culture-based methods or microscopy alone. In addition to limited sensitivity, culture-based diagnostics involving non-sterile samples suffer from limited specificity: Positive culture results can represent fungal colonization which can lead to misdiagnosis and overtreatment [58]. Nevertheless, fungal culture and microscopy (from sterile sample) are essential for detecting rare mold infections, such as mucormycosis, fusariosis, scedosporiosis, lomentosporosis, and infections caused by other rare molds, such as *Microascus* (formerly *Scopulariopsis*), *Rasamsonia*, or basidiomycetes. These pathogens are normally only detected by fungal culture (or microscopy) with subsequent ITS sequencing or MALDI-TOF MS [59] of the isolate for species identification.

#### 3.2.2. Antigen-Based Diagnostics

Although galactomannan (GM) detection plays a crucial role for the diagnosis of IA, several studies have shown that systematically screening for GM in blood for the detection of bIFI in patients receiving either posaconazole or micafungin during high-risk episodes is not useful due to the low prevalence of infection and the associated low positive predictive value of a positive test result [60,61]. As a consequence, the 2017 ESCMID–ECMM–European Respiratory Society (ERS) guidelines for the diagnosis and management of *Aspergillus* disease provides a recommendation against the use of serum GM screening in patients on mold-active prophylaxis [62]. However, antigen-based diagnostics remain critical to manage suspected fungal disease in these patients. Several studies demonstrated a better performance of GM detection in BAL versus blood, whereas BDG testing only provides reliable results in blood [63]. BAL GM testing may be particularly warranted in non-neutropenic patients, which often show an airway invasive growth pattern of IA, and therefore rarely produce positive serum GM results [64,65]. In a homogenous cohort of acute myeloid leukemia patients during induction chemotherapy, increasing the posaconazole concentration was shown to decrease the sensitivity of serum GM assay. In general, the sensitivity of serum GM assay to detect probable and proven IA is 81.8%, but none of patients with IA and a posaconazole concentration ≥ 0.5 mg/L had a positive serum GM test [66]. Slightly reduced sensitivities in the presence of mold-active antifungals has also been described for the BAL GM: at a cut-off of 0.5 optical density index (ODI), Eigl and colleagues showed a 71% sensitivity for probable/proven IA in those on antifungals versus 95% in those without antifungals [7,67,68]. Sensitivity for diagnosing breakthrough IA in patients on antifungal prophylaxis dropped to 52% in that study when using a 1.0 ODI cutoff [68]. Therefore, using a lower cut-off of 0.5 ODI from BALF for diagnosing breakthrough IA may be preferable and was also recommended in another study [67]. Combining several antigen detection assays or antigen detection tests with PCR has shown convincing diagnostic potential for the diagnosis of breakthrough mold infections [56,69,70]. Data on the performance of the new lateral flow tests for the diagnosis of breakthrough IA, such as the AspLFD (OLM Diagnostics, Newcastle upon Tyne, UK; herein LFD) and the sōna Aspergillus galactomannan LFA (IMMY, Norman, OK, USA; herein LFA), are limited. For the LFD prototype test, sensitivity in BALF was only 52% in those receiving mold-active antifungals versus 86% in those not receiving mold-active antifungals [68], with a similar impact shown for serum LFD results in an animal model [27]. For the LFA, so far limited data has shown no significant impact of mold-active antifungals on efficacy [57,71,72,73,74]. Thus, both the LFA and the LFD are attractive options for the diagnosis of breakthrough infections in BALF and serum, especially when used in combination with other biomarkers [75]. Mold-active prophylaxis is affecting the epidemiology of invasive mycoses resulting in a shift towards less common entities such as fusariosis. In a retrospective cohort (2004–2017) from a tertiary hospital in Madrid, Spain, all (*n* = 7) cases of breakthrough invasive fusariosis were characterized by positive BDG tests in blood [76], and GM testing has also been shown to be useful for diagnosing fusariosis [77]. Lastly, a combination of multiple antigen-based diagnostics, conventional diagnostics, PCR-based assays, and novel diagnostic markers can help to diagnose breakthrough mold infections. Particularly, combination of GM with one or more other tests, such as the LFA, LFD, or PCR-based assays, shows promise for diagnosis of breakthrough IA in case of clinical suspicion, with positivity of one of those tests indicating breakthrough IA, even when others result negative.

#### 3.2.3. Nucleic Acid-Based Assays/Others

*Aspergillus* PCR has demonstrated high sensitivity and high negative predictive value in severely immunocompromised patients in settings where antifungal prophylaxis is not used [78,79,80,81]. PCR is also an important diagnostic test for mucormycosis [82,83]. However, the performance of PCR from blood is impacted significantly in patients receiving mold-active antifungal agents as shown in a recent review [8,55,81,84]. While mold-active prophylaxis seems to affect *Aspergillus* PCR on BALF less than blood, reduced diagnostic performance has also been described from BALF [85]. Given the reduced sensitivity of all diagnostic tests in the presence of mold-active antifungal prophylaxis or treatment, the combination of multiple diagnostic tests is warranted [8,55,56,69,86]. Immunological markers may also be utilized as combination partners, and particularly high serum IL-8 levels (>300 pg/mL) have been shown to be highly specific for IA [56,87,88,89], and have shown high sensitivity and specificity when combined with BALF LFD or BALF *Aspergillus* PCR [56]. Larger multicenter studies are currently in progress to validate these findings. The most secreted siderophore of *A. fumigatus* is triacetylfusarinine C (TAFC), which is produced only by actively growing cells, is not present in conidia, and can be detected in urine, BALF and blood [90,91,92]. TAFC can be detected by mass spectrometry and has shown excellent performance as a biomarker for breakthrough IA in urine samples, when normalized to urine creatinine, with similar performance to those reported for GM determination in serum and BALF [90]. In BALF, TAFC was shown to be an independent biomarker for IA, improving the performance of BALF GM for detection of IA when used in combination [91]. These results warrant further exploration of this promising new biomarker. Other potential approaches for mycological detection of IA include the detection of volatile organic compounds in exhaled air [93], and Bis (methylthio) gliotoxin, an inactive derivative of gliotoxin [94].

#### 3.2.4. Consensus Recommendation

The diagnosis of breakthrough mold infections is challenging, as all diagnostic tests have reduced sensitivity in samples obtained during treatment or prophylaxis with mold-active antifungals.

- Culture, microscopy, and antifungal susceptibility testing are essential for the diagnosis of breakthrough mold infections, particularly for infections other than invasive aspergillosis. Cultures of the lower respiratory tract are mostly preferred, although blood cultures may be positive in some cases. If necessary, and susceptibility testing, particularly for mold infections other than IA. Blood invasive procedures to obtain a biopsy and definite proof of bIFI should be considered. Importantly, a negative fungal culture does not rule out a breakthrough invasive mold infection, given the low sensitivity of culture in this setting.
- Despite reduced sensitivities, antigen-based diagnostics, such as GM (in BALF and serum) and BDG (in serum only), or newer assays, such as LFA and LFD (both in BALF or serum), have important roles for diagnosing breakthrough IA when the degree of clinical suspicion is high, because the sensitivity of fungal culture may be even further reduced.
- While we do not recommend using these tests for screening in patients on mold-active prophylaxis or treatment, a combination of multiple antigen-based diagnostics, conventional diagnostics, PCR-based assays, and novel diagnostic markers can help to diagnose breakthrough mold infections.
- Many of the available antigen-based diagnostics such as GM or the LFA and the LFD tests are specific for IA and very few other mold infections such as fusariosis, therefore, negative test results do not automatically rule out a breakthrough mold infection, but instead should raise the suspicion for mucormycosis or another rare mold as a potential causative pathogen.

### 3.3. Diagnosis of Breakthrough Infections Due to Endemic Mycoses

#### 3.3.1. Conventional Diagnostics

Diagnosis of the endemic mycoses (*Blastomyces*, *Coccidioides*, *Emergomyces*, *Histoplasma*, *Paracoccidioides*, *Sporothrix* spp., and *Talaromyces marneffei* (formerly *Penicillium marneffei*) is confirmed by histopathologic or direct microscopy of specimens from an affected site. Samples obtained by bronchoscopy are most frequently examined following pneumonia or when suspicious lesions are identified on radiographic imaging. However, biopsy results of affected sites or cerebrospinal fluid are also frequently helpful if cultures or typical in vivo findings of these fungi are observed [44].

Culture provides confirmation of infection and allows for susceptibility testing or identification to the species level, although the clinical correlation of susceptibility results to clinical outcomes has not been definitively demonstrated for the endemic mycoses. However, in vitro MICs do suggest resistance likely occurs [95,96], may develop on therapy [97], and may be increasing in frequency [98,99]. With attempts at culture isolation, biosafety is an important consideration when handling these organisms, and laboratories should incorporate national guidance and regulations into their processes and practices to ensure the safety of laboratory staff.

#### 3.3.2. Serology

Serologic testing is widely used for the diagnosis and care of patients with coccidioidomycosis. In this group, serology has been found to be helpful diagnostically, but also correlates with patient symptoms and is useful to follow prognostically as a biomarker of infection. For example, relapse of infection in patients with coccidioidomycosis is typically accompanied by a rise in the complement fixation (CF) antibody titer [100]. Serology of blastomycosis is less helpful due to the lower sensitivity and specificity of testing [101,102,103]. Serologic testing for histoplasmosis utilizing antigen testing is most useful for patients with chronic pulmonary disease and may not be helpful in those with severe immunosuppression [104,105,106,107]. Still, in the right patient quantitative antigen methods with monitoring of *Histoplasma* antigen titers can allow for monitoring response during treatment [54]. In contrast, paracoccidioidomycosis serologies exhibit high sensitivity and specificity [108,109,110]. Sporotrichosis serologic testing is infrequently used due to the lack of a commercial assay, while the sensitivity of antibody testing for talaromycosis ranges from 30–80% likely due to the highly immunosuppressed state (e.g., advanced HIV disease) of most affected patients [111,112].

It is important to recall that in the immunosuppressed patient population the endemic mycoses, particularly *Coccidioides* or *Histoplasma*, may recur years after initial infection, and serology may not be positive or may have aberrant kinetics compared to immunocompetent hosts [100,113,114].

Prior to initiating immunosuppressive therapy, it is often prudent to evaluate a patient’s past travel history to determine the individual risk for endemic mycoses. For those with a suggestive history, serologic testing can be performed to ascertain the potential need or to guide prophylaxis/treatment practices to avoid potential breakthrough infection later.

Antigen testing for *Blastomyces* spp. is commercially available and has a reported sensitivity of 85–93% and a specificity of 79–99% [115,116,117,118,119]. Test positivity in *Coccidioides* depends upon the degree of host immunosuppression and is largely unhelpful in the immunocompetent. In highly immunosuppressed patients, antigenuria has been detected in up to 70% of patients [120]. *Histoplasma* antigen assays are most useful in patients who have disseminated histoplasmosis and acute pulmonary histoplasmosis, but are less useful in localized pulmonary infection and chronic cavitary pulmonary histoplasmosis [121,122]. Antigen detection for paracoccidioidomycosis has been investigated although is not commercially available [123], and has not been evaluated for sporotrichosis. Antigen detection in talaromycosis is highly accurate and is well suited for patients with advanced immunosuppression and a high blood fungal burden [124]. However, it is not widely available.

#### 3.3.3. Nucleic Acid Based Assays/Others

Nucleic acid amplification using polymerase chain reaction (PCR) tests are not commercially available for the endemic mycoses, but detection of DNA in clinical specimens has been evaluated for: *Blastomyces* (sensitivity 60–86%) [125,126,127], *Coccidioides* (~50%) [128,129], *Histoplasma* (18–65%) [106,130], *Paracoccidioides* (91–100%) [114,131] *Sporothrix* (83–92%) [132,133,134], and *Talaromyces* (70–86%) [135].

The use of BDG for the diagnosis of endemic mycosis is problematic due to the lack of specificity and the poor positive predictive value and although have been evaluated in limited fashion, are generally unhelpful in the diagnosis or management of endemic mycoses [136,137].

#### 3.3.4. Consensus Recommendation

Specific recommendations for diagnosis of breakthrough endemic mycoses include:

- Whenever possible, diagnosis of bIFI caused by endemic mycoses should be confirmed by obtaining affected tissue for examination by direct microscopy, histopathology, and fungal culture.
- More nuanced approaches are required for individual diseases that are suspected based on the relevant clinical picture and exposure history. In acute disease in immunocompromised patients, histoplasmosis and talaromycosis can both be diagnosed with antigen tests, although the latter assay is not widely available.
- Antibody tests for coccidioidomycosis, paracoccidioidomycosis, and acute and chronic histoplasmosis should be considered, but antibody tests for histoplasmosis are not recommended in patients with immunosuppression or those with cystic fibrosis. Serology for other endemic mycoses (i.e., blastomycosis, sporotrichosis, emergomycosis) have limited sensitivities and specificities or are not commercially available.

## 4. Discussion

The diagnosis of bIFIs remain challenging, with limited sensitivities of most available fungal diagnostics. With these consensus recommendations we intend to support the design of future clinical trials in the field of clinical mycology.

The diagnosis of breakthrough yeast infections and the endemic mycoses should rely on the isolation of the causative pathogen, such as by conventional culture methods, although the yield is often reduced in patients on antifungal prophylaxis, making diagnosis of bIFI even more challenging. In addition, while culture-based methods can enable species identification and antifungal susceptibility profiles to help guide antifungal treatment breakpoints, such as the minimum inhibitory concentration (MIC) that measure in vitro drug activity, do not always reliably predict in vivo drug activity and clinical outcome. For instance, the pharmacokinetic/pharmacodynamic properties of the drug, potential drug-drug interactions, and the overall health and immune status of the patient receiving the antifungal drug can all affect the in vivo activity of the drug in the human body [13]. Other non-culture methods such as BDG and PCR for the diagnosis of yeast infection and serologic tests for acute histoplasmosis and coccidioidomycosis can be considered, but the effect of antifungal therapy likely decreases the yield of these tests, although this has not been well-studied.

Conversely, antigen-based assays, such as GM, the LFD, and LFA, have an important role in the diagnosis of breakthrough invasive mold infections, although antifungal therapy may reduce the sensitivity of these assays and a combination of multiple antigen-based diagnostics, along with conventional culture and PCR-based assays, may further increase the diagnostic yield. Breakthrough infections occurring under antifungal prophylaxis mostly require combinations of multiple tests and biomarkers in order to achieve an acceptable sensitivity. Optimally, diagnostic approaches for fungal infections should be initiated before initiation of antifungal treatment, however in the real world this is often not possible.

More effective, simpler, and cheaper diagnostic tests are needed with more rapid turnaround time to diagnose bIFIs, particularly non-*Aspergillus* mold infections and endemic mycosis. Given that antifungal therapy can decrease the diagnostic yield of conventional culture and several serologic and PCR-based assays discussed, improved diagnostics, particularly for bIFIs, are needed. While these definitions represent the status of published literature, future studies are needed to fill important gaps.
